# Satisfaction with Information Provided to Infertile Patients Who Undergo Assisted Reproductive Treatment

**DOI:** 10.22074/ijfs.2019.5314

**Published:** 2018-10-02

**Authors:** Zahra Ezabadi, Fahimeh Mollaahmadi, Saeideh Sazvar, Samira Vesali, Reza Omani-Samani

**Affiliations:** 1 Department of Epidemiology and Reproductive Health, Reproductive Epidemiology Research Centre, Royan Institute for Reproductive Biomedicine, ACECR, Tehran, Iran; 2 Department of Endocrinology and Female Infertility, Reproductive Biomedicine Research Centre, Royan Institute for Reproductive Biomedicine, ACECR, Tehran, Iran

**Keywords:** Awareness, Health Promotion, Infertility, Information, Satisfaction

## Abstract

**Background:**

Potentially modifiable factors, such as the appropriate informing process given to infertile patients, can affect their infertility knowledge and information. This study aims to assess infertility information provided to Irani- ans who undergo assisted reproductive treatment.

**Materials and Methods:**

In this cross-sectional study, participants recruited were a convenience sample of all infer- tile patients who received assisted reproductive treatments from Royan Institute, Tehran, Iran. Inclusion criteria con- sisted of: patient’s first time visit, no previous infertility treatment failures, and referral to the centre between January and March 2015. A 20-item tool designed by researchers measured patient satisfaction with the infertility informing process. This tool included cause of infertility, type of recommended treatment, diagnostic procedures, approximate treatment duration, success rate of the treatment, approximate cost of treatment, and non-therapeutic factors in treat- ment success.

**Results:**

A total of 235 infertile patients were invited to participate in the study, from which 200 (100 men and 100 women) participants completely responded to the questionnaire with a response rate of approximately 85%. The mean age of participants was 30.93 ± 5.56 years. In terms of satisfaction with information provided about the cause of in- fertility, male responders reported the lowest mean score of 3.59 ± 1.05 compared to female responders (3.82 ± 0.85, P=0.078). Infertile women had a greater mean score of 3.85 ± 0.78 than infertile men (3.58 ± 1.29) in satisfaction with information provided about the type of recommended treatment (P=0.037). There was a statistically significant difference between males (3.26 ± 1.04) and females (3.58 ± 0.93) in satisfaction with approximate treatment duration (P=0.031).

**Conclusion:**

According to the results, most infertile patients were satisfied with the informing process related to the cause of infertility and recommended therapies. Information about infertility should be provided more systemati- cally to all treated patients by medical staff, especially in terms of success rate of treatment and financial cost of therapy.

## Introduction

Infertility is defined by the failure to achieve a clinical pregnancy after 12 months or more of regular unprotected sexual intercourse (1). Infertility is a biomedical health problem and a reproductive system disease or dysfunction (2). Some potentially modifiable factors, such as provision of appropriate information to infertile patients, can affect infertility knowledge and information seeking behaviour among people who undergo assisted reproductive treatment.

Health-seeking behaviour among couples with infertility is directly related to their understanding of reproductive biology and their beliefs about infertility. Those with better knowledge of fertility health issues may show improved use of health care resources with a consequent reduction in infertility (3, 4).

Infertility awareness is considered a critical first step towards fertility preservation or infertility care by lifestyle modifications or changes (1, 5). Because fertility knowledge is associated with education, recommended health promotion strategies should begin with educational interventions (2). Fertility health promotion represents that knowledge is a key factor associated with fertility self-care and the initiation of treatment. Focusing on education about fertility issues is needed to prevent fear and unnecessary delay in seeking help when faced with problems in conception (5).

Providing relevant information to patients, respecting their wishes, and considering their capacity to make treatment decisions is crucial for high-quality and patient-centred fertility care (2). Satisfaction with care can originate from adequate patient education, which enables patients to give greater understanding of and participation in medical decision-making, and often results in better health outcomes (6). There is an enormous disparity in the literature about the perspective of the infertile clients’ satisfaction with infertility information provided by medical staff. Relatively little is known about satisfaction with information provided to infertile patients who receive infertility treatment. This study is the first in Iran to examine satisfaction with information provided to infertile patients who undergo assisted reproduction treatment.

## Materials and Methods

This descriptive cross-sectional study was the first phase of a large survey on women and men who undergo infertility treatment in the largest referral fertility clinic in Iran, Royan Institute, where people are examined from all socio-economic and ethnic backgrounds. Participants recruited were a convenience sample of all infertile patients who received first-time assisted reproductive treatments, and who did not have any previous infertility treatment failures. Patients were seen at Royan Institute between January and March, 2015.

In this questionnaire-based study, the researchers developed a tool that was validated on the basis of a literature review. The questionnaire included questions about satisfaction with information about cause of infertility (3 questions); type of recommended treatment (3 questions); diagnostic procedures (3 questions); approximate treatment duration (3 questions); success rate of the treatment (3 questions); approximate financial cost of treatment (3 questions); and non-therapeutic factors in treatment success such as diet, exercise, taking supplements, and cigarette smoking (2 questions) to measure satisfaction with infertile patients’ self-perception of the informing process.

Demographic and clinical information of the participants were gathered from their records in the fertility centre. Question types included yes/no, a 5-point Likert scale that ranged from 1 to 5 (dissatisfied, low satisfaction, neither satisfied nor dissatisfied, satisfied, very satisfied), and choice of one option. The questionnaire was also designed for the Iranian context and validated by a group of 18 gynaecologists, embryologists, methodologists, and nurses for content, ease of understanding, and acceptability. Face validity was performed by a Persian literature expert and the wording of the questions was adapted to the context and perspectives of the participants. One interviewer who was aware of the main objective of the present study was responsible for distribution and collection of the questionnaires among participants.

The Ethics Committee of Royan Institute approved this study (EC/92/106). Aims of the study were clearly explained for all participants prior to the investigation. Voluntarily completion of the questionnaire was considered as consent. Eligible individuals were assured that their confidentiality and anonymity, and that their decision to participate in or withdraw from the study would not impact their current or future relationship with the clinic. Participants were also assured that their level of satisfaction did not affect provision of care services.

Statistical analyses were carried out using the Statistical Package for Social Science (SPSS, version 20.0 for Windows; SPSS Inc., Chicago, IL, USA). Continuous variables were expressed as mean ± SD (standard deviation) and categorical variables as numbers (percentages). Responses with the 5-point Likert scale (range: 1 to 5) were compared by the independent samples t test because it is robust when one might encounter ordinal scaled data. The statistical issue was demonstrated by Heeren and D'Agostino (7) in 1987 as previously explained. P<0.05 was considered statistically significant.

## Results

In this study, 235 infertile patients were invited to participate. In total, 200 participants (100 men and 100 women) responded to the questionnaire completely and returned completed questionnaires, which yielded a response rate of about 85%. The mean age of participants was 30.93 ± 5.56 years. Of participants, 67 (33.5%) patients were diagnosed with male infertility and 31 (15.5%) had female infertility. Recommended therapies to the patients included the following: 36 (18%) *in vitro* fertilization (IVF), 52 (26%) microinjection, and 71 (35.5%) intrauterine injection (IUI). Among diagnostic procedures used for the participants, 95 (47.5%) patients were diagnosed by ultrasound and 114 (57%) by blood and urine tests. Table 1 lists the demographic characteristics of the infertile study participants.

A total mean score of satisfaction with the informing process for each area in Table 2. As shown, the highest mean scores of satisfaction with the informing process to patients were related to cause of infertility (3.71 ± 0.96) and recommended therapies (3.72 ± 0.91). The lowest mean score satisfaction with informing process to the patients was 3.31 ± 1.10 for the approximate financial cost of treatment.

**Table 1:** Demographic and clinical characteristics of the study participants (n=200)


Demographic and clinical variables	n (%)

Sex	
Male	100 (50)
Female	100 (50)
Cause of infertility	
Male	67 (33.5)
Female	31 (15.5)
Both	54 (27)
Unknown	36 (18)
No answer	12 (6)
Recommended therapies	
In vitro fertilization (IVF)	36 (18)
Micro injection	52 (26)
Intra uterine injection (IUI)	71 (35.5)
Other	5 (2.5)
No answer	22 (11)
Diagnostic procedures	
Hysteroscopy	14 (7)
Ultrasound	95 (47.5)
Blood and urine	114 (57)
Laparoscopy	7 (3.5)
Pap smear	41 (20.5)
Genetic counseling	10 (5)
Hysterosonography	4 (2)
Hystrosalpangiography	39 (19.5)
Sperm motility	73 (36.5)
No answer	30 (15)


**Fig.1:**
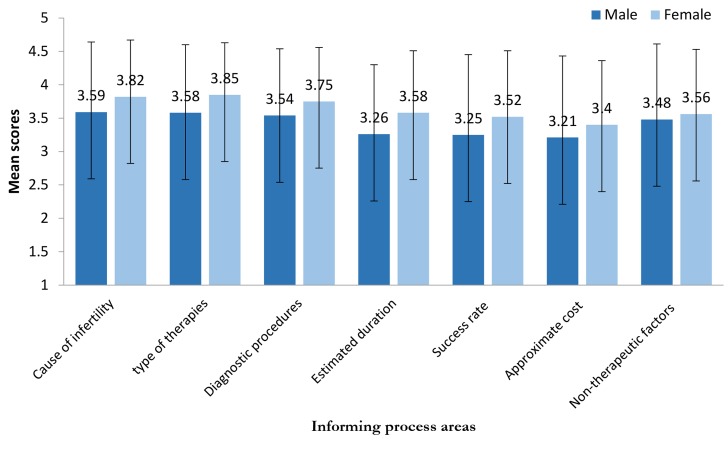
Patients’ satisfaction with the informing process areas (n=200 participants).

In terms of satisfaction with information provided about cause of infertility, male responders reported the lowest mean score (3.59 ± 1.05) compared to female responders (3.82 ± 0.85); there was no statistically significant difference between men and women (P=0.078, Fig .1). Infertile women had a statistically greater mean score (3.85 ± 0.78) than infertile men (3.58 ± 1.29) in satisfaction with information provided about type of recommended treatment (P=0.037). A statistically significant difference was observed between males (3.26 ± 1.04) and females (3.58 ± 0.93) in satisfaction with approximate treatment duration (P=0.031). More than half of the responders obtained their infertility treatment information (causes, therapies, diagnostic procedures, cost, duration, and success rate) from physicians instead of other medical staff (P<0.001, Table 3).

**Table 2:** Description of infertile patients’ satisfaction with the informing process


Areas	Mean	Standard deviation	Minimum	Maximum

Cause of infertility	3.71	0.96	1	5
Recommended therapies	3.72	0.91	1	5
Diagnostic procedures	3.64	0.91	1	5
Estimated treatment duration	3.42	0.99	1	5
Success rate of the treatment	3.39	1.11	1	5
Approximate cost of treatment	3.31	1.1	1	5
Non-therapeuticfactors in treatment success	3.52	1.5	1	5


**Table 3:** Frequency of information on infertility treatment obtained from medical staff


Areas	Medical staff				
	Physiciann (%)	Nurse n (%)	Midwifen (%)	Receptionn (%)	Other n (%)

Cause of infertility	127 (67.2)	9 (4.2)	17 (9.0)	13 (5.8)	23 (12.2)
Recommended therapies	117 (65.0)	9 (5.0)	16 (8.9)	11 (5.6)	27 (15.0)
Diagnostic procedures	126 (71.6)	10 (5.7)	6 (3.4)	15 (8.5)	16 (9.1)
Estimated treatment duration	103 (60.6)	17 (10.0)	11 (6.5)	12 (7.1)	24 (14.1)
Treatment Success rate	92 (58.2)	20 (12.7)	18 (11.4)	6 (3.8)	19 (12.0)
Approximate cost of treatment	72 (47.7)	12 (7.9)	6 (4.0)	25 (16.6)	33 (21.9)
Non-therapeutic factors in treatment success	94 (58.8)	12 (7.5)	7 (4.4)	18 (11.3)	27 (16.9)


## Discussion

To the best of our knowledge, this was the first national survey of infertile clients that pertained to satisfaction with information provision in infertility care. Determining the extent of the patients’ satisfaction with information about infertility and its treatment would be beneficial for planning education programs related to the prevention of failures in infertility treatment or withdrawal. The findings of this study have provided useful insights into potentially modifiable factors that influence infertile patient’s co-operation with medical staff in the infertility clinics and compliance with assisted reproductive treatments.

From this study, it was apparent that most infertile patients who participated were more satisfied with the informing process related to the cause of infertility and recommended therapies. In contrast, the vast majority of participants were less satisfied with the information provided for approximate financial cost of their treatment. Overall satisfaction with this infertility care centre was usually high in the survey, but provided no reliable measure for the quality of care (8-10). There were considerable knowledge gaps, particularly in relation to the impact of infertility treatment failure and infertility treatment history in other fertility care clinics.

Of note, those who had a history of infertility treatment were more aware of infertility-related information such as causes, therapeutic procedures, and financial cost. Hence, over-reporting of satisfaction with information provided to the patients was unavoidable. In this study, we attempted to recruit all infertile patients who received assisted reproductive treatments for first time and did not have any previous infertility treatment failures.

Problems exist with the absence of data registration from all Iranian infertility care clinics. However, objective data collection on satisfaction with information provision in infertility care is difficult. This study was in line with most studies that relied on interviews. Possibly, the answers of the respondents were to some extent biased by incorrect recall and self-interest (10, 11). Another limitation of this study was the obvious gender bias towards women in our sample; all women studied acquired higher scores of satisfaction with information provided in all 7 domains compared to the male respondents. It was likely that most infertile women have used the Internet for information on fertility-related problems. Men would be less likely to seek services for infertility than women, and many men from infertile couples do not undergo a male examination (12). Selection bias might occur against people who have a low income or a migration background, which is a common finding in interview studies on assisted reproductive treatment (8, 10).

To the best of our knowledge, we did not find any research that measured satisfaction with infertility treatment information available to infertile people in the literature. Rauprich et al. (10) investigated the views of patients (n=1590) and experts, including physicians (n=230) and psychosocial counsellors (n=66), in Germany on information provision and decision-making in assisted reproduction treatment. Most participants had positive views for information on the chances for treatment success and physical risks of fertility treatment than for information on the risks and burden of multiple pregnancies, and on the emotional risks and burden associated with infertility treatment.

In the present study, both men and women participants were more satisfied with information provided about type of recommended treatment. The objective of another study was to assess patients’ satisfaction with the investigation and initial management of infertility in 1366 women who attended outpatient clinics at 12 hospitals throughout Scotland. Overall, 87% of respondents were satisfied or very satisfied with their care, but there were a number of deficiencies identified.

A total of 86% felt they had not been given enough assistance with the emotional aspects of infertility, whereas 47% felt they were not given a clear plan for the future and 23% of those who had been given drug treatments reported receiving little or no information about the treatment or possible side-effects (11). In the present study, more than half of responders received their infertility information from physicians instead of other medical staff. A qualitative study with 6 group discussions on fertility knowledge and information-seeking behaviour among people of reproductive age revealed that most women and men who intended to have children in the future agreed that primary health care providers, such as general practitioners (GPs), were well placed to provide information regarding fertility and pregnancy health (13).

Despite the remaining limitations and risks of bias, the present methodical strategies have provided sufficient validity for the principal results of the study. The findings are limited to the particular context of fertility care in Iran, and are not transferable or generalizable elsewhere.

## Conclusion

Information about infertility should be provided more systematically to all treated patients by medical staff, especially for success rate of treatment and financial cost of therapy. However, most infertile patient participants were more satisfied with the informing process related to the cause of infertility and recommended therapies. Therefore, the information should be clarified for all infertile patients prior to the onset of any therapeutic procedures.
